# Effectiveness of a smartphone-based educational intervention to improve breastfeeding

**DOI:** 10.1186/s13006-021-00417-w

**Published:** 2021-09-20

**Authors:** Navisa Seyyedi, Leili Rahmatnezhad, Maryam Mesgarzadeh, Hamidreza Khalkhali, Negisa Seyyedi, Bahlol Rahimi

**Affiliations:** 1 Student Research Committee, School of Allied Medical Sciences, Urmia University of Medical Sciences, Urmia, Iran; 2 School of Allied Medical Sciences, Urmia University of Medical Sciences, Urmia, Iran; 3 School of Nursing and Midwifery, Urmia University of Medical Sciences, Urmia, Iran; 4 School of Public Health, Urmia University of Medical Sciences, Urmia, Iran; 5 Nursing Care Research Center, Iran University of Medical Sciences, Tehran, Iran

**Keywords:** mHealth, Educational early intervention, Breastfeeding, The study was registered in the Iranian Registry of Clinical Trials (IRCT Id: IRCT20190705044103N2) at 25 September 2020

## Abstract

**Background:**

Exclusive breastfeeding (EBF) is essential during the first six months of life and confers countless benefits to mothers and infants. This study aimed to assess the effectiveness of a smartphone-based educational intervention to improve new mothers’ breastfeeding for infants younger than six months of age in Urmia, Iran.

**Methods:**

A randomized controlled trial study was conducted from January to December 2019 with 40 new mothers and their first child aged < 3 months, assigned to the intervention (mobile app education + routine care) and control groups (routine care). The mean age of infants was 1.25 and 0.98 months for each group consequently. The designed app content categorized according to seven sections (the importance of breastfeeding, behavioral methods, complementary feeding and EBF, pumping and manual expression, managing common breast-related and breastfeeding problems, breastfeeding tips in special situations, and common queries) for educating the required knowledge to nursing mothers.

**Results:**

Forty mothers were assessed for primary outcomes in each group. At three months, the mothers’ knowledge, attitude, and practice (KAP) had meaningful differences in the intervention group compared to the control group. In the intervention group, the degree of changes in knowledge and attitude were 5.67 ± 0.94 and 8.75 ± 1.37 respectively more than the control group (*p* < 0.001, p < 0.001). However, this amount for the practice score was 0.8 ± 0.49 which is considered to be marginally significant (*p* = 0.063). During the study, the mothers’ breastfeeding self-efficacy showed significant progress in favor of the intervention group. The score enhancement was 26.85 ± 7.13 for the intervention group and only 0.40 ± 5.17 for the control group that was confirmed to be significant (p < 0.001).

**Conclusion:**

The smartphone-based app for educating new mothers on breastfeeding had a significantly positive effect on breastfeeding self-efficacy and maternal KAP. In future studies, the intervention can be tested in both prenatal and postpartum periods.

## Background

New mothers encounter several breastfeeding challenges [1]. There are numerous factors influencing the beginning and continuation of breastfeeding, and some of the leading predictors are maternal intention to breastfeed, knowledge deficiency about the improvement of lactation, and little confidence in possessing breastfeeding skills [2, 3]. Meanwhile, in several studies, maternal breastfeeding self-efficacy has been strongly associated with EBF duration [4, 5].

The implementation of health education programs greatly contributes to promoting maternal breastfeeding knowledge, attitude, and practice (KAP) [6–8]. Parents’ education is associated with an awareness of proper child-rearing practices, better health-seeking attitudes, improved knowledge about breastfeeding/complementary feeding, and up-to-date information about immunization [8, 9]. By conducting four 60-min sessions of an educational program, Kang et al. reported remarkable improvement rates in breastfeeding empowerment and practice [10].

Healthcare professionals and researchers have introduced mobile health (mHealth) as an effective technological solution for promoting healthy living, access to health information, chronic disease monitoring, and patients’ self-management [11–13]. By investigating health information-seeking behaviors among perinatal women, Osma et al. confirmed that these women chiefly use the Internet and mobile devices for such purposes, and also commonly download health-related apps on their smartphones [14]. However, due to increasing concerns about the poor quality of infant-feeding commercial apps, the necessity of research-tested smartphone apps to provide reliable information about the health benefits of breast milk has been emphasized in previous studies [15].

In the past decade, the Ministry of Health and Medical Education in Iran has made great efforts to provide educational materials such as breastfeeding booklets or pamphlets. Still, due to a lack of support from health providers, these collections are not successfully delivered to mothers and, therefore, breastfeeding still has low rates in Iran [16]. To the best of our knowledge, no trial has evaluated the effectiveness of a smartphone app for educating mothers on breastfeeding in Iran. Thus, this study aimed to assess the impact of an educational intervention based on a smartphone app for improving breastfeeding during the infants’ first six months of life in Urmia, Iran.

## Methods

### Study setting

A randomized controlled trial (RCT) with one educational intervention arm (smartphone app + routine care) and one control arm (routine care) was conducted from January to December 2019 in Urmia, Iran. The Research Ethics Board of Urmia University of Medical Sciences approved the study design (approval code: IR.UMSU.REC1396.105).

### Eligibility and recruitment

The participants were selected from a well-child clinic in Urmia. Mother-infant pairs who visited this clinic were screened by midwives other than those in the research team. Based on the inclusion criteria, new mothers who intended to breastfeed and had their firstborn child aged < 3 months were selected. Women were excluded if they did not have a smartphone, or if the infant had a physical disability or cerebral palsy. We also excluded mothers and infants whose physician had recommended formula feeding due to breastfeeding contraindications because of the mothers’ health condition (e.g., positive T-cell lymphotropic virus, untreated brucellosis, varicella, H1N1 influenza). If the cases met the inclusion criteria, the healthcare provider ensured that the mothers were inclined to breastfeeding based on an oral interview, and then invited the mothers to talk to a research assistant about the study. In the next step, a consent form was signed; the mother’s contact details were obtained; and a questionnaire pack, including demographic details, KAP, and self-efficacy measuring queries, was completed.

### Randomization and blinding

After baseline assessment, eligible mothers were allocated to the intervention or control group using a Web-based randomization tool [17] with a 1:1 ratio. Data collectors and care providers were blinded to group assignment and just investigated the inclusion criteria in the clinic. The researcher implementing the randomization procedure was blind to the participants and had not cooperated in the recruitment process. After determining intervention and control group members, telephone calls were made to the mothers in the intervention arm and they were asked to install the mHealth app-based educational program sent to their phones over the Internet and social media. Alternatively, they could visit the clinic and have the app installed on their phones. We visited the intervention group in a different room to avoid the control group’s possible access to the mobile app.

### Description of the intervention

The app was designed to provide the required knowledge for educating nursing mothers based on the Ministry of Health and Medical Education’s Maternity Guideline for Maternal and Child Health [18]. The content used for the education program had seven sections: the importance of breastfeeding, behavioral methods for mothers, complementary feeding and EBF, pumping and manual expression, managing common breast-related and breastfeeding problems, breastfeeding tips in special situations (e.g., for infants with Down syndrome, cleft lip, or cleft palate), and answering common queries about lactation in case of illness.

Due to the importance of a participatory design during the development process for creating a user-friendly tool [19], we adopted a collaborative partnership model comprising a health-promotion software developer, two childcare experts, three medical informaticists, and end-users, in a revising procedure. Finally, an Android-compatible app was developed due to significant community interest in Android-based smartphones, and was released for the use of the intervention group.

In both intervention (smartphone-based education + routine care) and control groups (routine care), mother-infant pairs were visited in the health center by a health service provider. During this routine care, the infants were vaccinated and assessed on developmental aspects based on weight, height, and screening tests to determine their abilities and overall health. They also had a face-to-face session for their monthly routine visits during the intervention.

Moreover, in the intervention arm, the app was installed on the mothers’ phones. This app was developed in a topic-oriented educational manner to facilitate discovering the solution to problems encountered through the lactating process. The intervention was scheduled for a three-month period. Two midwives other than those in the research team followed-up the mothers as the directly responsible parent and encouraged them to use the app by calling them once a week. During the follow-ups, the mothers were asked about their breastfeeding problems and were recommended to refer to the different sections of the app.

### Data collection

The process of data collection was conducted from January to December 2019. During each pre- and post-intervention appointment, the questionnaire pack consisting of maternal breastfeeding self-efficacy, KAP, and demographic questions was completed by the mothers. The study focused on the following data: (1) sociodemographic information on the infants’ age and sex, mothers’ age, family income, education, and occupation; and (2) mothers’ KAP, as well as self-efficacy of breastfeeding. The first part of this questionnaire was based on a standard scale named the Breastfeeding Self-Efficacy Scale-Short Form (BSES-SF) [20, 21], and the second part (KAP) was designed based on the smartphone educational app’s contents, as well as several previous studies [22–27].

Randomization was performed with a 1:1 ratio between the intervention and control group. A total sample size of 80 mother-infant pairs was estimated based on an 80% statistical power at a two-tailed α of 0.05. In a previous study, the standard deviation of knowledge was 4.39 [28], and we aimed for 2.7 differences in mean score of knowledge between intervention and control groups. Therefore, considering a 20% dropout rate, the total sample size was planned to be 100 (50 per group).

### Data analysis

First, we compared the baseline and follow-up characteristics of the participants in the intervention and control groups by applying chi-square tests and an independent t-test for mothers’ age. The criterion for significance was set at α = 0.05. All the statistical analyses were conducted in SPSS, version 16. To investigate group differences, independent t-tests were performed. In addition, the results were compared after the intervention too. Finally, the changes between baseline and post-intervention in each TAU and smartphone group were differentiated to show the effectiveness of the intervention more clearly. Analysis was by intention-to-treat and participants were analyzed according to their randomized assignment.

## Results

### Characteristics of infants and mothers

**Fig. 1:**
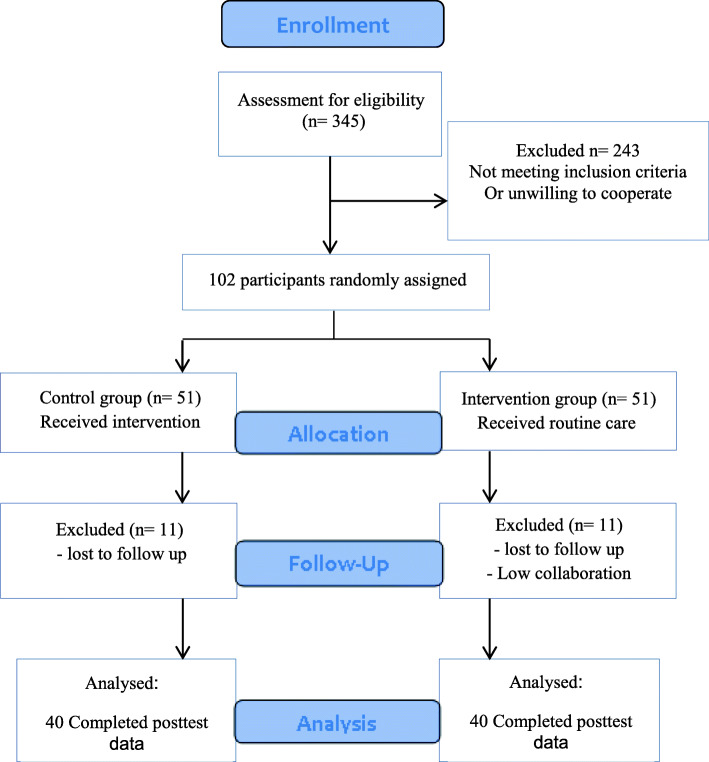
CONSORT diagram describing study enrolment and allocation

Eighty mother-infant pairs completed both before and after surveys in the two groups. The participants were recruited from January to June 2019, and the intervention period continued until October 2019. Figure 1 displays a flowchart of the participants’ characteristics. Randomization produced groups with no statistically significant differences in socio-demographics in terms of the mothers’ age, education, occupation or economic status, however the residency was marginally significant (*p* = 0.08). Table 1 presents the baseline characteristics of the participants with a mean age of about 26 years, of whom 30% had an academic education, 75% were homemakers, 51.25% had a middle-high income status, and 71.25% lived in rural areas. The infants’ (53.8% girls, 46.2% boys) mean age was 1.25 months for the intervention and 0.98 for the control group. There was no significant difference in the infants’ age between the two groups (*p* = 0.206).

**Table 1:** Characteristics of participating mothers

Data		Intervention ***n*** = 40 n (%)	Control n = 40 n (%)	***P*** Value
**Education**	Middle school	13 (32.5)	15 (37.5)	*P* = 0.33
	Diploma	12 (30)	16 (40)	
	Academic	15 (37.5)	9 (22.5)	
**Job status**	Employed	3 (7.5)	5 (12.5)	*P* = 0.15
	Home employed	9 (22.5)	3 (7.5)	
	House wife	28 (70)	32 (80)	
**Income status**	Cost>Income	17 (42.5)	22 (55)	*P* = 0.23
	Cost = Income	15 (37.5)	15 (37.5)	
	Cost<Income	8 (20)	3 (7.5)	
**Residency**	City	2 (5)	3 (7.5)	P = 0.08
	City margin	5 (12.5)	13 (32.5)	
	Village	33 (82.5)	24 (60)	

### Efficacy of the intervention

#### Impact of the mobile app on mothers’ KAP

At baseline, we confirmed the homogeneity of the two groups based on their pretest scores using the independent t-test. Accordingly, mothers’ knowledge, practice and attitude were similar (*p* = 0.128, *p* = 0.954 and *p* = 0.095; see Table 2). Table 2 compares the pre- and post-test scores obtained upon administering the surveys on mothers’ KAP by group. After the follow-up, the mothers’ knowledge was significantly higher in the intervention group (*p* < 0.001). Similarly, attitude and practice scores were significantly improved (*p* = 0.010, *p* = 0.033) among mothers in the intervention group in comparison to the control group. Consequently, the overall rate of KAP was significantly higher in the experimental group than in the control group (*p* < 0.001).

**Table 2:** Mothers’ KAP statistics results in the intervention and control groups

	Mothers’ KAP scores mean (95% confidence interval).					
	***Baseline***			***3-month follow-up***		
	Intervention n = 40	Control n = 40	***P***-value	Intervention n = 40	Control n = 40	P-value
**Knowledge**	13.15 (11.52, 14.78)	14.63 (13.62, 15.64)	0.128	20.23 (19.27, 21.19)	16.03 (15.03, 17.03)	< 0.001
**Attitudes**	50.55 (46.92, 54.18)	54.25 (51.77, 56.73)	0.095	65.35 (62.39, 68.31)	60.30 (57.53, 63.07)	0.010
**Practice**	7.43 (6.71, 8.15)	7.45 (6.96, 7.94)	0.954	9.13 (8.7, 9.56)	8.35 (7.78, 8.92)	0.033
**KAP score**	71.13 (65.75, 65.75)	76.32 (73, 79.64)	0.104	94.70 (91.38, 98.02)	84.67 (80.85, 88.49)	< 0.001

*KAP* knowledge, attitude, practice

Based on Table 3, there was weak evidence for the difference in practice pre-test and post-test scores between the groups (*p* = 0.063). However, the degree of change in knowledge and attitude significantly differed between the two groups. Furthermore, the score of KAP demonstrated a significant change with a 23.57 ± 5.09 enhancement in the intervention group (*p* < 0.001).

**Table 3:** Post-intervention – pre-intervention changes in mothers’ KAP scores in the intervention and control groups

	Post-intervention – pre-intervention change mean (95% confidence interval).			
	Intervention n = 40	Control n = 40	Between-group difference	P-value
**Knowledge**	7.07 (5.37, 8.77)	1.40 (0.64, 2.16)	5.67 (4.82, 6.61)	< 0.001
**Attitudes**	14.80 (11.42, 18.18)	6.05 (4.04, 8.06)	8.75 (7.38, 10.12)	< 0.001
**Practice**	1.70 (0.85, 2.55)	0.90 (0.54,1.26)	0.8 (0.31, 1.29)	0.063
**KAP Score**	23.57 (18.48, 28.66)	8.35 (5.6, 11.1)	15.22 (12.47, 17.97)	< 0.001

#### Impact of the mobile app on maternal breastfeeding self-efficacy

The mothers’ breastfeeding self-efficacy score was also similar between the groups at baseline (*p* = 0.281; see Table 4). The intervention group made remarkable progress during the study period. The pooled difference in the reported indices between the groups favored the intervention group over the control group. The mothers’ self-efficacy in the intervention group was enhanced by about 26.85 ± 7.13, but that of the control group increased by only 0.40 ± 5.17. The change significantly differed between the two groups (p < 0.001; see Tables 4 and 5).

**Table 4:** Mothers’ breastfeeding self-efficacy statistics results in the intervention and control groups

	Mothers’ breastfeeding self-efficacy scores mean (95% confidence interval)					
	***Baseline***			***3-month follow-up***		
	Intervention n = 40	Control n = 40	P-value	Intervention n = 40	Control n = 40	P-value
**Self-efficacy**	119.08 (111.27, 126.89)	125.10 (117.17, 133.03)	0.281	145.92 (140.14, 151.70)	125.50 (117.17, 133.83)	< 0.001

**Table 5:** Post-intervention – pre-intervention changes in mothers’ breastfeeding self-efficacy scores in the intervention and control groups

	Post-intervention – pre-intervention change mean (95% confidence interval)			
	Intervention n = 40	Control ***n*** = 40	Between-group difference	P-value
**Self-efficacy**	26.85 (19.72, 33.98)	0.40 (−4.77, 5.57)	26.45 (24.49, 28.41)	< 0.001

## Discussion

This study assessed the effect of smartphone-based maternal education on breastfeeding among primiparous mothers in terms of maternal KAP, as well as self-efficacy of breastfeeding. The results demonstrated larger improvement scores for the intervention group compared to the control group. Based on a recent Iranian systematic review, any education or advice received from influential sources can improve the mean score of self-efficacy in a moderate or strong manner [29, 30]. An earlier study evaluated the impact of an educational program and reported a significant difference (*p* < 0.01) for breastfeeding self-efficacy mean scores between intervention and control groups [31]. Our study, however, resulted in a greater change in this parameter than did the mentioned study. This could be explained by the dedication of limited sessions to educating mothers, including only one 2.5-h session in the last month of pregnancy, and telephone counseling twice within two weeks after childbirth [31].

In the present study, the majority of the participants were highly interested in receiving evidence-based educational materials for the better growth of their children. Some of them reported that they had searched for online information but could not find sufficient information. There are concerns about most of the health-related apps on the market in terms of using insufficient evidence-based contents, and these apps may not deliver accurate information to users [32–35]. Some studies on breastfeed-promoting mobile apps have focused on their acceptability and usability measures which need more investigation on their effectiveness [36].

Similar results have been found in different studies reporting the highest impact of breastfeeding education during pre- and postnatal periods [37] and, in some cases, placing more emphasis on prenatal education as an initial action [38, 39]. However, due to time limitations, the present study could support only the postpartum educational intervention, which merits examination in longer durations in future studies.

An RCT has recently been published by Wu et al. [40] about the effectiveness of a breastfeeding empowerment mHealth intervention on EBF along with antenatal and postnatal care. The study reported the intervention group’s EBF rate at the 0–1-month postpartum follow-up to be significantly higher (*p* < 0.001) than that of the control group, but in the later months the difference between the two groups gradually declined. They explained the reason for this by the social and cultural pressure on mothers to introduce formula or to wean the infant after the second month of birth. Although our study has not examined this point of view, it has investigated the practical aspect of mothers’ breastfeeding. As mentioned in the Results section, we found mothers’ practice to be less affected than other aspects, and this reminds us to pay more attention to restricted multichannel supports and follow-ups. Another RCT has been designed by Doan et al. [41] in a similar way to evaluate the effectiveness of a breastfeeding mobile phone app for improving the EBF rate. This study is still in progress and will publish the results in the future.

Based on a previous review recommendation, a multicomponent intervention can be more effective than a single-method intervention [42]. Dennis et al. designed a mobile app with three modules, including tutorials, station locators, and a milk donor platform to empower breastfeeding, which had high acceptability among mothers [43]. Nevertheless, the intervention in our study was mainly educational with consultation in some cases using social media and phone call follow-ups, and resulted in significant effectiveness. Still, more attention to counselor pairing may boost the intervention’s efficacy.

Several studies have reviewed the importance of social networks’ causal effects on health behaviors. The use of a collaborative setting among the interested parties such as social networks could enhance individuals’ motivation and skills for adopting healthy behaviors [8, 44–46]. The findings of some review studies on educational apps for nursing mothers also indicated that social support, which is underdeveloped in many of the applications, is a major influencing feature on breastfeeding duration [47, 48]. A recent Iranian study proposed an educational intervention about breastfeeding and complementary feeding based on the Telegram social network and reported 85.4% satisfaction among the mothers [49]. In our study, the use of social networks was limited to the participants’ follow-up and individual connection with two midwives; it would be better to employ group capabilities such as discussion forums and share personal experiences with more maternal involvement.

Parents tend to use programs if they are recommended by health professionals or a recognized government body [50]. Thus, to further enhance effectiveness, national organizations’ co-operation with project implementation is of utmost importance.

This study had some limitations. First, the restricted time permitted us to implement only a three-month intervention for the postnatal period. Secondly, the app was programmed only for Android users, albeit very few people use iOS. Other limitations include the low recruitment rate which was only 30% of 345 assessed mother-child pairs.

## Conclusion

By designing a smartphone-based app for mothers with a topic-oriented and problem-based educational approach, this study helped mothers discover solutions during breastfeeding. The results indicated a considerable improvement in mothers’ KAP. Furthermore, this intervention strongly affected mothers’ breastfeeding self-efficacy. Therefore, this educational intervention using mobile apps can be useful for mothers in breastfeeding. The effectiveness of educational smartphone-based apps for both prenatal and postpartum management merits evaluation by future studies.

## Data Availability

The data used and/or analyzed during the current study are available from the corresponding author on reasonable request.

## Abbreviations

App: Application
BSE: Breastfeeding Self-Efficacy
BSES-SF: Breastfeeding Self-Efficacy Scale-Short Form
EBF: Exclusive breastfeeding
KAP: Knowledge, attitude, and practice
